# Mass-Forming Portal Biliopathy Presenting as Extreme Wall-Thickening of the Common Bile Duct

**DOI:** 10.3390/diagnostics10090623

**Published:** 2020-08-22

**Authors:** Jan Binne Hulshoff, Frans J. C. Cuperus, Robbert J. de Haas

**Affiliations:** 1 Department of Radiology, University of Groningen, University Medical Center Groningen, P.O. Box 30 001, 9700 RB Groningen, The Netherlands; jb.hulshoff@umcg.nl; 2 Department of Gastroenterology, University of Groningen, University Medical Center Groningen, P.O. Box 30 001, 9700 RB Groningen, The Netherlands; f.j.c.cuperus@umcg.nl

**Keywords:** portal biliopathy, portal vein thrombosis, cholangitis, cholangiocarcinoma

## Abstract

Portal biliopathy refers to biliary tree abnormalities in patients with peribiliary collateral vessels and non-neoplastic extrahepatic portal vein occlusion. These biliary abnormalities are caused by vascular compression and ischemic damage of the biliary tree, which can result in bile duct compression, stenosis, fibrotic strictures, bile duct dilation, and thickening of the bile duct wall. Portal biliopathy is difficult to distinguish from cholangiocarcinoma, IgG4-related disease, and sclerosing cholangitis. Although most patients are asymptomatic, portal biliopathy can lead to serious complications, such as recurrent cholangitis. This case illustrates the importance of including portal biliopathy in the differential diagnosis at an early stage, especially in patients with portal hypertension. With early recognition, the need for additional invasive diagnostic procedures such as biopsies is minimized. Pathogenesis, clinical presentation, diagnostics, and treatment options of portal biliopathy are described in the article.

## 1. Introduction

Portal biliopathy is caused by direct vascular compression and ischemic damage to the biliary tree in patients with cavernous transformation of the portal vein caused by non-cirrhotic/non-neoplastic chronic extrahepatic portal vein occlusion [1]. Although most patients with biliary dilation due to portal biliopathy remain asymptomatic, jaundice, choledocholithiasis, and recurrent cholangitis do occur [2]. Portal biliopathy mimics several other entities, such as cholangiocarcinoma or (auto-immune and sclerosing) cholangitis. Therefore, it is important to be aware of the clinical presentation and imaging characteristics of portal biliopathy to prevent unnecessary biopsy or surgery.

Our case illustrates that magnetic resonance imaging (MRI) combined with MR cholangiopancreaticography (MRCP) and complementary endoscopic ultrasound (EUS) facilitate the recognition of portal biliopathy. A timely diagnosis, however, remains challenging because portal biliopathy can be a ‘great mimicker’ of cholangiocarcinoma, sclerosing cholangitis, and IgG4 cholangiopathy.

## 2. Case Report

A 52-year-old Afro-Caribbean man with a history of hypertension presented at our tertiary referral center with longstanding abdominal pain in the right upper quadrant and unintentional weight loss of 10 kg in 12 months. The pain started immediately after eating, and worsened when eating food with high amounts of fat. The patient did not complain of having fatty stools or diarrhea. His physical examination was unremarkable. Laboratory investigations revealed increased levels of alkaline phosphatase (168 U/L; reference <115 U/L), gamma-glutamyl transferase (244 U/L; reference <55 U/L), and total bilirubin (27 μmol/L; reference <17 μmol/L). There was no history of alcohol use or cigarette smoking, and the family history was negative for relevant diseases. At presentation, our patient had been unemployed for several years. Our patient has provided written informed consent.

**Figure 1:**
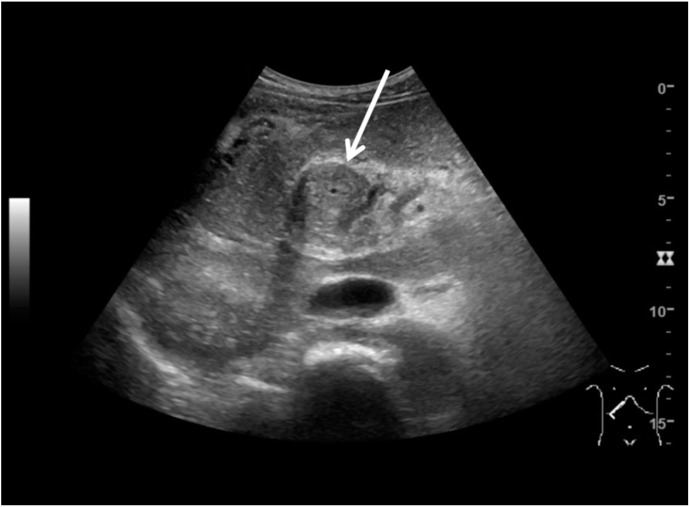
Ultrasound image of the abdominal mass (5.9 × 8.7 cm; arrow) in the right upper quadrant, surrounding the common bile duct.

**Figure 2:**
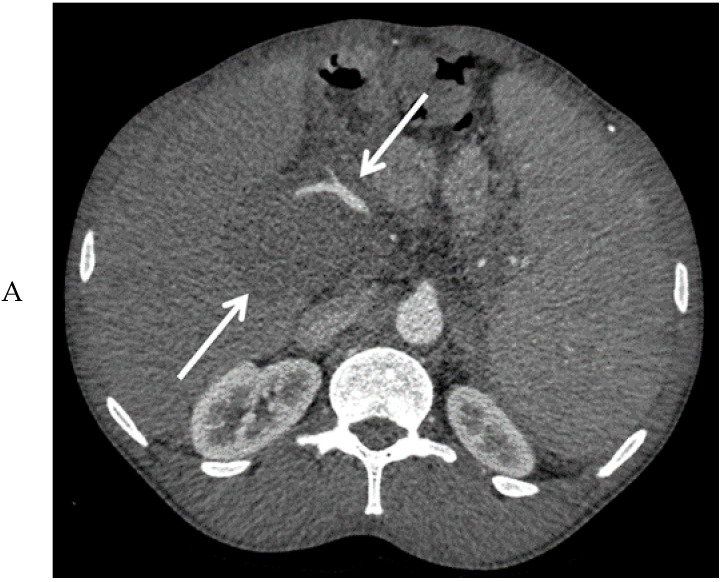

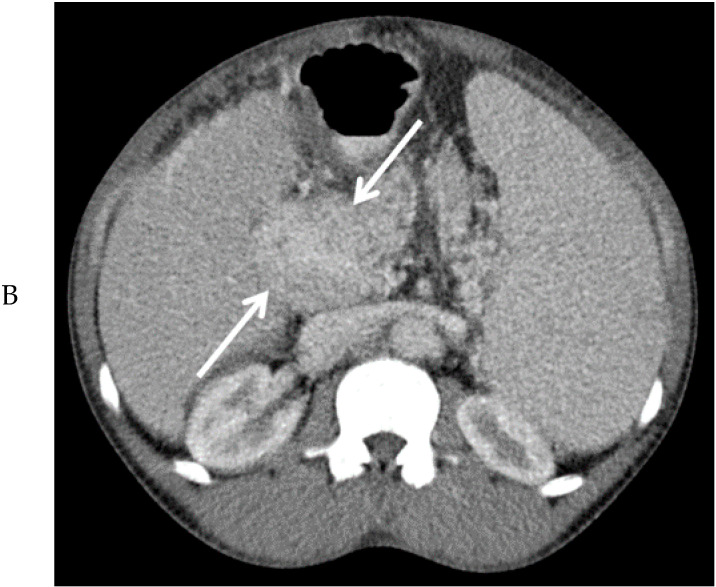
Computed tomography images of the mass in the upper abdomen (arrows) in the arterial (**A**) and portal venous phase (**B**).

Abdominal ultrasound showed a large mass between the liver hilus and the pancreatic head (Figure 1), an enlarged spleen, and extensive collateral vessel formation. Contrast-enhanced computed tomography (CT) confirmed the presence of this mass with encasement of the hepatic arteries and common bile duct, dilation of the intrahepatic bile ducts, and portal vein thrombosis (Figure 2A,B). Due to the vascular encasement, the mass was deemed irresectable. An esophago-gastroduodenoscopy showed large esophageal varices. Elastography (FibroScan) showed only moderate (F2) liver fibrosis.

To exclude a neuroendocrine tumor, Gallium-68 DOTATOC and 18F fluorodeoxyglucose positron emission tomography (PET) scans were performed, which showed no pathologic uptake. Follow-up CT after three months demonstrated stable disease. Tumor markers (alpha fetoprotein and carbohydrate antigen 19-9) and IgG4 levels remained within normal limits. IgG4 quantitative polymerase chain reaction (qPCR), however, showed a value of 13.9% (reference <5%), compatible with IgG4 disease of the pancreas and biliary tract [3]. Prednisolone treatment (40 mg/d, 6 weeks) for IgG4 disease, however, did not decrease the size of the mass at follow-up CT. 

**Figure 3:**
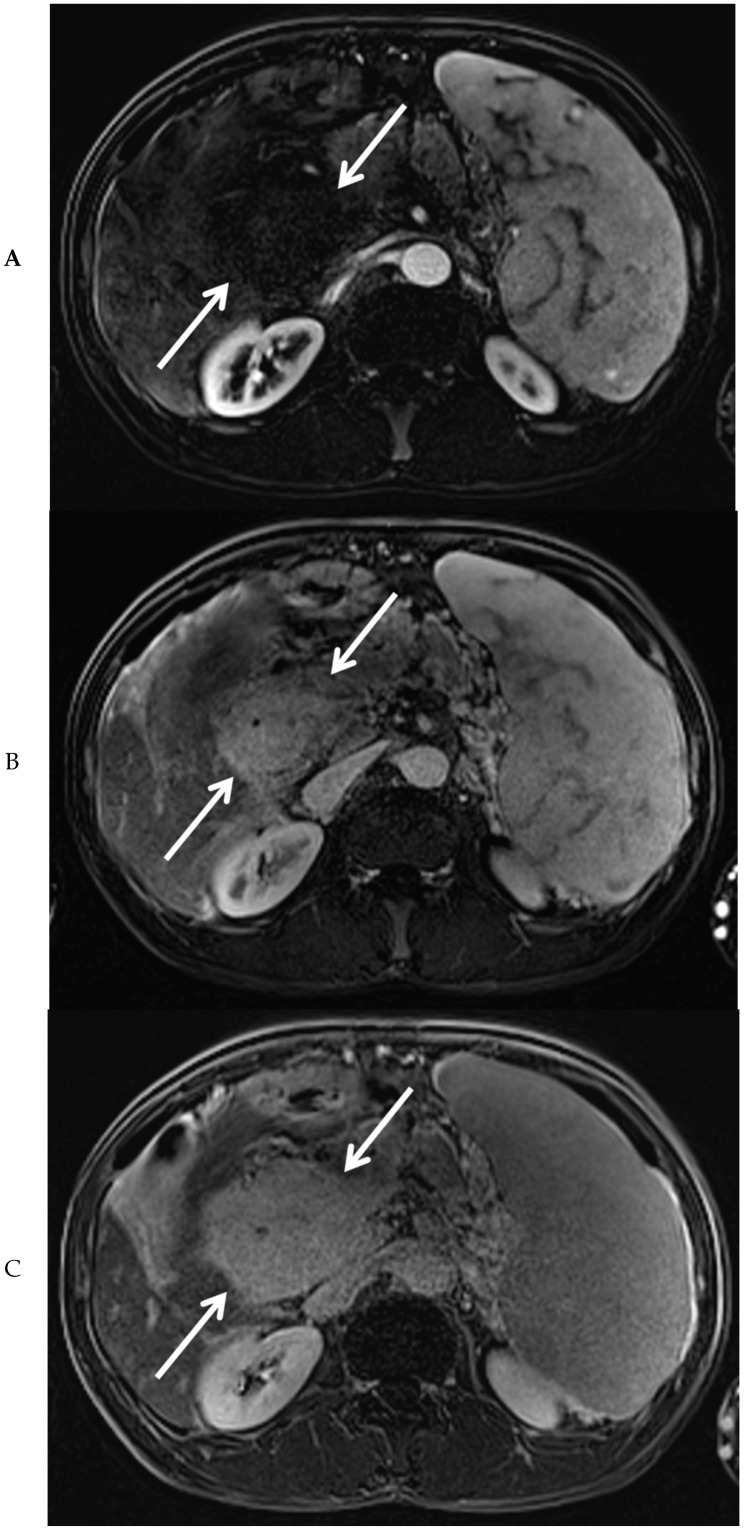

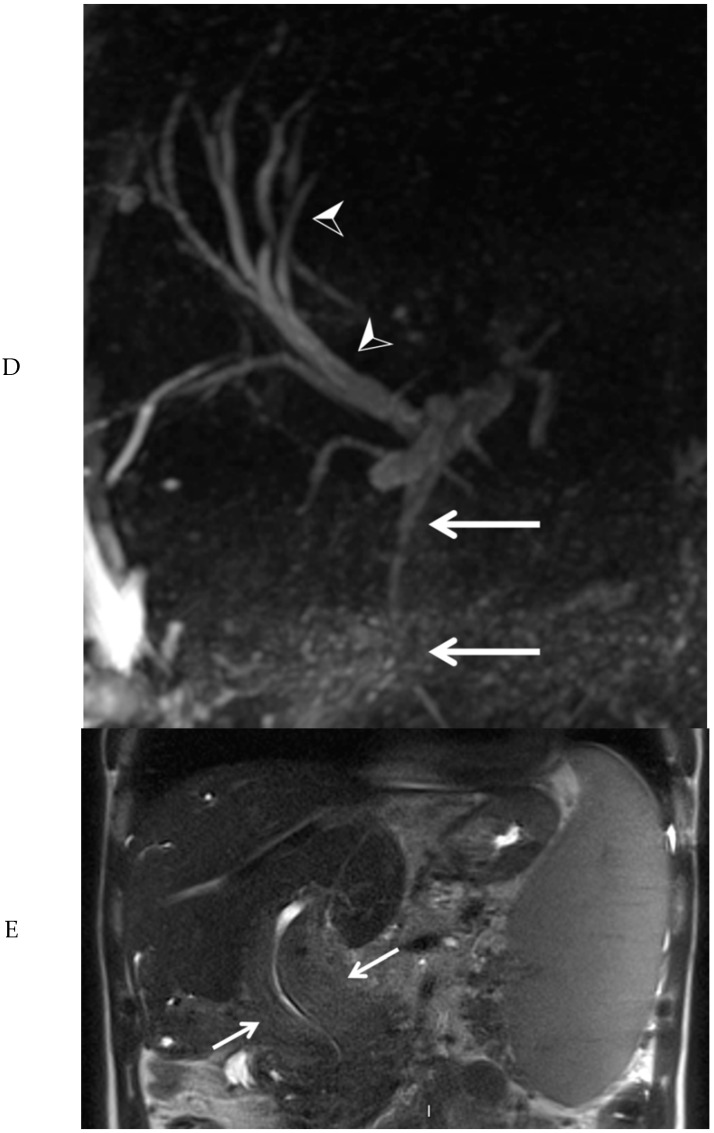
Magnetic resonance imaging of the mass in the upper abdomen (arrows). Shown are T1 weighted subtraction images of the mass in the upper abdomen with fat suppression after administration of intravenous contrast medium in the arterial (**A**), portal venous (**B**), and delayed phase (**C**). At magnetic resonance cholangiopancreaticography, narrowing of the common bile duct is visible along its complete course (arrows), combined with dilation of the intrahepatic bile ducts (arrowheads) (**D**). At T2-weighted imaging (**E**, coronal view), the mass surrounding the narrowed common bile duct was slightly hyperintense, when compared to the liver parenchyma (arrows).

Subsequently, EUS combined with fine needle biopsy, and MRI of the upper abdomen were performed. EUS showed a highly vascular mass with a benign aspect. EUS-guided fine needle biopsy was somewhat difficult due to hardness of the mass. The histopathologic findings were most consistent with a lesion of vascular origin, such as hemangioma. MRI of the upper abdomen revealed a large mass surrounding the common bile duct, central intrahepatic bile ducts, cystic duct, and gallbladder, without diffusion restriction. After administration of intravenous contrast medium, no enhancement of the mass was visible in the arterial phase (Figure 3A). However, many small vessels were visible within the mass in the portal venous phase (Figure 3B), with diffuse homogeneous enhancement of the mass in the delayed phase (Figure 3C). At the MRCP, narrowing of the common bile duct was visible along its complete course, combined with dilation of the intrahepatic bile ducts (Figure 3D). At T2-weighted imaging, the mass around the narrowed common bile duct was slightly hyperintense when compared to the liver parenchyma (Figure 3E).

After combining the findings during biopsy, at histopathology, and at all imaging modalities, the large mass in the upper abdomen can be explained by an extreme manifestation of mass-forming portal biliopathy. Additional analysis revealed a JAK2V617F mutation. This mutation leads to the development of myeloproliferative neoplasms, a common underlying cause of non-cirrhotic portal vein thrombosis [4]. Histopathological analysis after bone marrow biopsy confirmed the presence of polycythemia vera in our patient.

Currently, our patient is in good condition. However, he has experienced several episodes of iron-deficiency anemia, without signs of active bleeding in an esophago-gastroduodenoscopy. The iron-deficiency anemia is most probably caused by chronic blood loss in the digestive tract, and he is now being treated with oral iron therapy.

## 3. Discussion

After combining all diagnostic information, the large mass was ultimately diagnosed as an unusual manifestation of portal biliopathy. Because of the JAK2V617F mutation, the patient was more prone to develop portal vein thrombosis, which caused extensive collateral vessel formation surrounding the common bile duct, resulting in portal biliopathy. The main learning point in the present case is that clinicians should know that portal biliopathy can present with a large mass surrounding the bile ducts, especially in patients with a high risk of thrombotic events. If we had thought of this possibility in an earlier stage, it would have been unnecessary to perform extensive diagnostic exams (such as PET scans, EUS, and biopsies). In particular, when carefully evaluating the MR images, it is clear that there are numerous small collateral vessels in the thickened wall of the common bile duct, indicating the presence of collateral vessels due to chronic portal vein thrombosis, leading to portal biliopathy. Thus, our case underlines the importance of being aware of this diagnosis, especially in patients with portal hypertension, not only to prevent unnecessary biopsies and surgery, but also to shorten the period of diagnostic uncertainty for the patient.

Portal biliopathy is defined as the presence of biliary abnormalities in patients with non-cirrhotic/non-neoplastic extrahepatic portal vein obstruction [1,2]. After a (chronic) extrahepatic portal vein thrombosis, collaterals develop to bypass the obstruction, resulting in cavernous transformation of the portal vein. These collaterals, which involve the paracholedochal and epicholedochal venous plexuses and cholecystic veins, cause extrinsic compression of the intra- and extrahepatic bile ducts. In addition, underlying inflammatory and ischemic changes can result in peribiliary fibrosis [5]. The ischemic changes seem to be related to deficient portal blood supply of the bile ducts caused by portal vein thrombosis and cavernous transformation of the portal vein, and to thrombosis of small bile duct venules, resulting in stricture formation and fibrosis [6]. As in our patient, excessive deposition of connective tissue around the bile ducts due to ischemia can lead to an extreme mass surrounding the bile ducts [7]. In a slightly higher number of patients, biliary strictures seem to be caused by mechanical obstruction, however, both mechanical compression and ischemic damage can play a role in the pathogenesis of portal biliopathy [1,8].

Several studies have shown that bile duct changes occur in 81–100% of patients with extrahepatic portal vein obstruction, while only 5–30% of patients with portal biliopathy present with symptoms of biliary obstruction [5]. Clinical presentation includes abdominal pain, jaundice, recurrent cholangitis, and cholecystitis [1,9]. Secondary biliary cirrhosis can develop in the case of prolonged bile duct obstruction [10]. 

Portal biliopathy can be difficult to distinguish from cholangiocarcinoma, IgG4-related disease, and sclerosing cholangitis. As illustrated by the present case, MRI and MRCP are important for diagnosis, differential diagnosis, and follow-up. At MRCP, biliary stenosis, angulations, dilations, choledochal varices, and lithiasis can be observed [1]. The optimal radiological imaging consists of MRCP combined with contrast-enhanced MRI, thereby not only showing bile duct changes, but also offering simultaneous visualization of the portal vein and collaterals [11]. In this way, important information is obtained regarding the biliary and vascular abnormalities, which can guide the decision of the most optimal treatment strategy. In case of longstanding portal vein thrombosis, the portal vein is often difficult to visualize at CT or MRI due to fibrosis combined with collateral vessel formation, which was also seen in our patient. Besides MRI/MRCP, EUS can be performed which sometimes offers a more detailed evaluation of the biliary stenosis or mass, and can visualize pericholedochal venous collaterals [12]. Also, EUS can be combined with fine needle biopsy, such as was performed in our case. Endoscopic retrograde cholangio-pancreatography (ERCP) has no diagnostic role anymore, because of its invasive character with associated risk of complications. Currently, in portal biliopathy patients ERCP is reserved for therapeutic procedures only. To narrow down the differential diagnosis, tumor markers and IgG4 level should be determined, and CT and/or PET-CT scans might be indicated.

Asymptomatic patients do not require any treatment. Treatment of symptomatic patients with portal biliopathy should be individualized, with the aim of diminishing portal hypertension, and to treat biliary strictures. The majority of patients need multiple treatments during their life, but a consensus on the timing and priority of treatments is still not available [1].

## 4. Conclusions

Portal biliopathy can present with a large mass in the upper abdomen, due to extensive peribiliary mass-forming fibrosis caused by the development of collateral veins and inflammatory and ischemic changes after chronic portal vein thrombosis. It is an important diagnosis to be aware of, as it can mimic cholangiocarcinoma and cholangitis, which have their own treatment strategies and thus have to be excluded before making the diagnosis of portal biliopathy. By understanding the clinical presentation and diagnostic characteristics, patients will receive the best treatment, minimizing the need for risky biopsies and surgical treatments, and shortening the period of diagnostic uncertainty for patients. As can be learned from our case, in patients presenting with clear signs of portal hypertension, combined with a peribiliary mass with homogeneous delayed enhancement, mass-forming portal biliopathy should be considered the most likely diagnosis.
